# Endogenous Carbamylation of Renal Medullary Proteins

**DOI:** 10.1371/journal.pone.0082655

**Published:** 2013-12-26

**Authors:** J'Neka S. Claxton, Pablo C. Sandoval, Gary Liu, Chung-Lin Chou, Jason D. Hoffert, Mark A. Knepper

**Affiliations:** Epithelial Systems Biology Laboratory, National Heart, Lung and Blood Institute, National Institutes of Health, Bethesda, Maryland, United States of America; Emory University, United States of America

## Abstract

Protein carbamylation is a post-translational modification that can occur in the presence of urea. In solution, urea is in equilibrium with ammonium cyanate, and carbamylation occurs when cyanate ions react with the amino groups of lysines, arginines, protein N-termini, as well as sulfhydryl groups of cysteines. The concentration of urea is elevated in the renal inner medulla compared with other tissues. Due to the high urea concentration, we hypothesized that carbamylation can occur endogenously within the rat inner medulla. Using immunoblotting of rat kidney cortical and medullary homogenates with a carbamyl-lysine specific antibody, we showed that carbamylation is present in a large number of inner medullary proteins. Using protein mass spectrometry (LC-MS/MS) of rat renal inner medulla, we identified 456 unique carbamylated sites in 403 proteins, including many that play important physiological roles in the renal medulla [Data can be accessed at https://helixweb.nih.gov/ESBL/Database/Carbamylation/Carbamylation_peptide_sorted.html]. We conclude that protein carbamylation occurs endogenously in the kidney, modifying many physiologically important proteins.

## Introduction

Protein carbamylation is a post-translational modification that occurs in the presence of urea. In aqueous solution, urea is in equilibrium with ammonium cyanate (200∶1 molar ratio) [1]. Cyanate ion is known to carbamylate amino groups found on lysine and arginine side chains and the N- termini of proteins [2]:
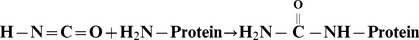
Additionally, it reacts with sulfhydryl groups found on cysteine side chains [2], [3]:
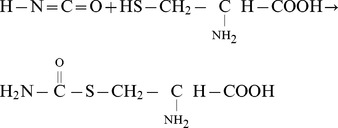



Several proteins have been found to be carbamylated in a variety of tissues: aquaporin-0 [4], αB-crystallin [5], apoA-I [6], insulin [7], and TIMP-2 [8].

The renal inner medulla accumulates urea at high concentrations, typically greater than 500 mM during antidiuresis [9], [10]. This raises the possibility that inner medullary proteins may be endogenously carbamylated due to exposure to high urea concentrations. Here, we address the question of whether there is widespread protein carbamylation in the rat renal medulla. To address this question, we used two approaches: 1) immunoblotting with a carbamyl-lysine specific antibody; and 2) large scale protein mass spectrometry of inner medullary proteins. The data showed extensive carbamylation of proteins within the renal inner medulla of rat. The mass spectrometry analysis identified 403 carbamylated proteins as well as the sites of carbamylation in their amino acid sequences.

## Methods

### Animals and Samples

All studies were done in male Sprague Dawley (SD) rats (250–350 g) and approved by the NHLBI Animal Care and Use Committee (Protocol Number: H-0110R2).

#### Animals for immunoblotting

In one set of studies, rats were water deprived for eight hours, receiving a dDAVP injection (2 nmol intraperitoneally (IP)) initially and a dDAVP injection (2 nmol intramuscularly) at five hours. Kidneys were rapidly resected and dissected into the following regions: cortex (outer), cortex (inner), outer medulla (outer), outer medulla (inner), inner medulla(outer), and inner medulla (inner). In an additional set of studies, rats were either water restricted (17 ml of water/day) or water loaded (40 ml of water/day) for five days. Kidneys were rapidly resected and the whole inner medulla was removed for processing.

In all experiments, animals were euthanized by decapitation without drug treatment. Samples were homogenized (Potter-Elvehjem homogenizer on ice, 30 strokes) in a 250 mM sucrose/10 mM triethanolamine (pH 7.6) solution.

#### Animals for mass spectrometry

For mass spectrometry studies, rats were injected with 5 mg furosemide (IP) thirty minutes before sacrifice to lower tissue urea. These animals were neither water restricted or given a vasopressin analog. After sacrifice by decapitation, kidneys were rapidly resected and inner medullas were isolated. Samples were homogenized (Potter-Elvehjen homogenizer on ice, 30 passes) in a 250 mM sucrose and 10 mM triethanolamine (pH 7.6) solution.

### Immunoblotting

Total protein content was determined with the BCA Protein Assay Kit (Thermo Scientific, Rockford, IL). Samples were then diluted with sample buffer (5X Laemmli buffer) at 20% (v/v) of the entire sample volume. Proteins were resolved by SDS-PAGE (4–20% polyacrylamide gels, Criterion, Bio-Rad, Hercules, CA) and transferred electrophoretically onto nitrocellulose membranes as previously described [11]. Membranes were probed with a goat polyclonal antibody that targets carbamylated lysines in proteins (Cell Biolabs, Inc., San Diego, CA, Catalog Number: STA-077) [12]. The antibody was used at 1∶500 dilution (in Odyssey blocking buffer containing 0.1% Tween 20) overnight at 4°C. After 1-h incubation with secondary antibody (Li-Cor Biosciences 680 anti-goat immunoglobulin G; Lincoln, NE) at 1∶5000 dilution, sites of antibody-antigen reaction were detected using an Odyssey infrared imager (Li-Cor).

### Mass Spectrometry

**Figure 1:**
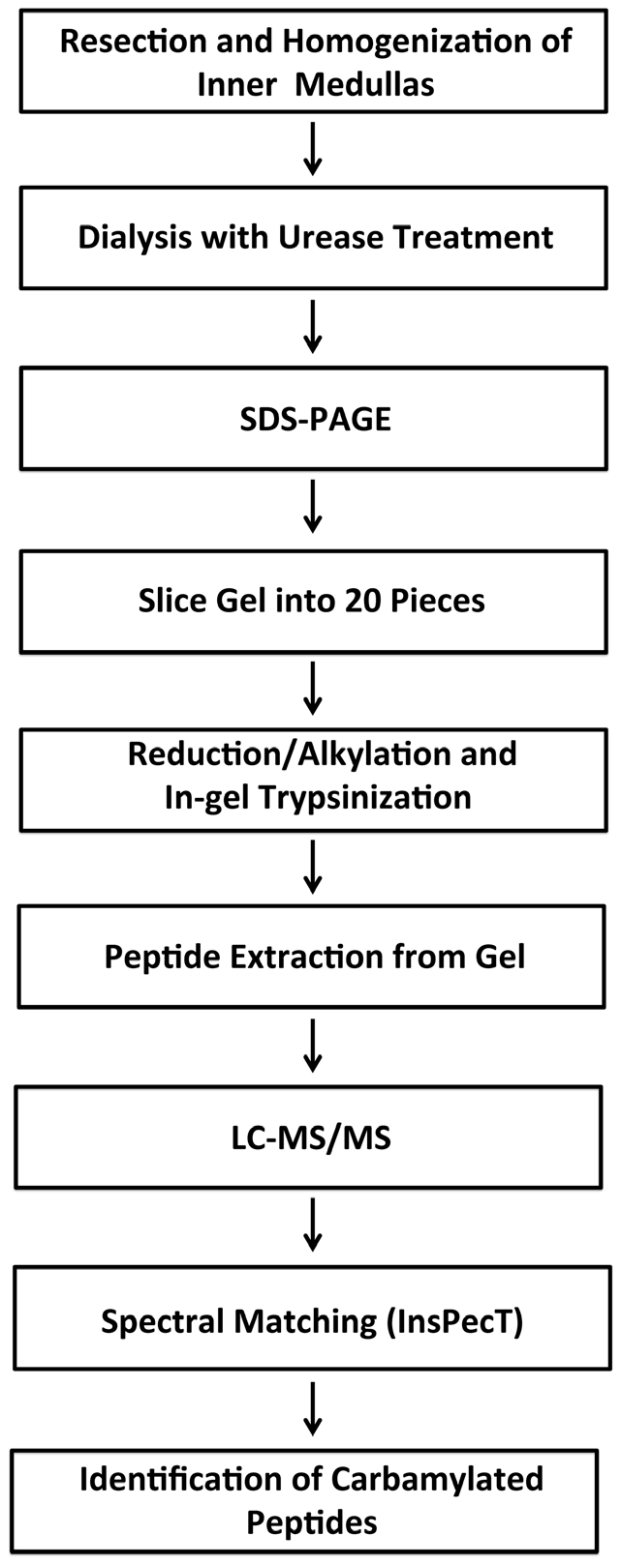
Workflow for mass spectrometry experiments.

The workflow for the mass spectrometry experiments is shown in Fig. 1. Renal inner medullary samples were passed through QIAshredder Mini Spin Columns (QIAGEN, Valencia, CA) to fragment DNA. Next, samples were dialyzed (3.5K M.W.C.O. Slide-A-Lyzer dialysis cassette, Thermo Scientific) for two hours at room temperature with urease (type III from *Canavalia ensiformis*, Sigma- Aldrich, St. Louis, MO) in the dialysis buffer at a concentration of 15,000 units/g. The dialysis was repeated for an additional 22 h at 4°C in the absence of urease. The total protein content of the dialyzed sample was then determined with the BCA Protein Assay Kit (Thermo Scientific). Samples were diluted in 5X Laemmli sample buffer as described above.

Each lane was equally loaded with 150 µg of protein for SDS-PAGE (4–20% polyacrylamide, Bio-Rad, Catalog Number: 345-0034). One lane was loaded with molecular weight markers (Bio-Rad, Catalog Number: 161-03775). Individual lanes were sliced with a razor blade according to the molecular weight markers to obtain 20 pieces. The gel slices were subjected to in-gel trypsin digestion, reduction, alkylation, and extraction from the gel as previously described [13].

The samples were analyzed on an Agilent 1100 nanoflow LC system (Agilent Technologies, Santa Clara, CA) connected to an LTQ Orbitrap Velos mass spectrometer (Thermo Scientific, San Jose, CA). Spectra were matched to specific rat protein sequences using *InsPecT* [14], a “hybrid” algorithm that uses partial *de novo* sequencing combined with pattern matching. The raw data files were searched against a concatenated forward and reverse database that included the most recent RefSeq rat protein database from the National Center for Biotechnology Information (NCBI) appended with a list of common contaminants. Carbamylated peptides were identified by the presence of a +43.005814 Da modification of lysine, arginine, cysteine, or any N-terminus. The peptides identified were sorted from best to worst using the *P* value parameter in *InsPecT*. The *P* value threshold was selected to provide a one percent false positive identification rate on a peptide level.

### Pseudo Immunoblot of Water Loaded and Water Restricted Rats

SDS-PAGE (4–20% polyacrylamide, Bio-Rad, Catalog Number: 345-0034) gels were equally loaded with 150 µg of protein. Individual lanes were sliced with a razor blade using the molecular weight marker (Bio-Rad, Catalog Number: 161-03775) as a guide to obtain 40 pieces. The gel slices were subjected to in-gel trypsin digestion, reduction, alkylation, and extraction from the gel as previously described [13].

The samples were analyzed on an Agilent 1100 nanoflow LC system connected to an LTQ Orbitrap Velos mass spectrometer as described above. Spectra were matched to specific rat protein sequences using *Sequest* [15]. Relative quantification of peptides was performed using QUOIL software [16]. Using the quantification data, peptide intensities for individual carbamylated peptides were summed over each of the 40 slices. To generate a heat map, we assigned values from 0–255 (8-bit gray scale) for each slice scaled to the maximum intensity found, oriented by molecular weight from top to bottom in the manner of a standard immunoblot using in-house software to generate the heat map images. Zero values (no peptides detected) were assigned a gray scale value of 5 in order to contrast with the surrounding page. Values above zero were scaled linearly.

### Bioinformatics

*Automated Bioinformatics Extractor* (ABE, http://helixweb.nih.gov/ESBL/ABE/) was used to extract Gene Ontology terms from the NCBI database as described by Hoffert *et al* [17]. *ProMatch* [18] was used to assign peptides to corresponding protein isoforms. This program is available at http://helixweb.nih.gov/ESBL/ProMatch/.

## Results and Discussion

### Analysis of Protein Carbamylation by Immunoblotting

**Figure 2:**
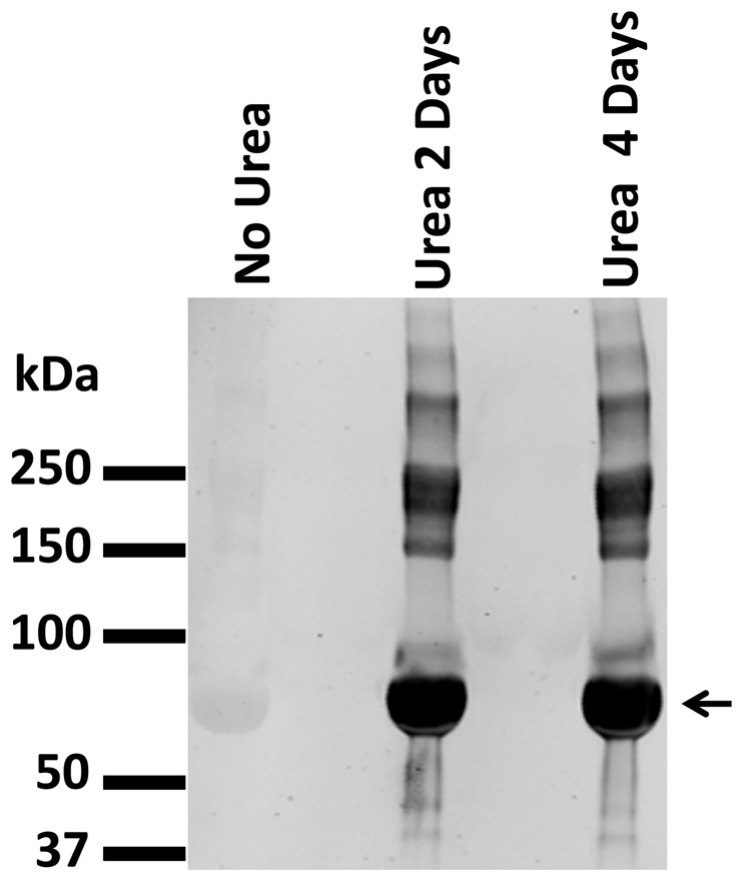
Specificity of anti-carbamyl-lysine antibody. Bovine serum albumin (BSA, 3 mg/ml, MP Biomedicals, Solon, OH) was incubated in 1M urea (in PBS) or PBS alone for two or four days. Following incubation, samples were dialyzed using 10,000 MWCO centrifugal filter devices (Microcon, Millipore, Billerica, MA). Samples were immunoblotted as described in *Methods*. Arrow indicates BSA monomer. Control sample used was incubated for four days in PBS alone.

Anti-carbamyl-lysine antibodies have been in use for many years [12]. We used a commercially available anti-carbamyl-lysine antibody to address whether carbamylated proteins are present in the kidneys of rats. As a preliminary confirmation of the specificity of the antibody, we incubated bovine serum albumin with 1 M urea (a concentration similar to that present normally in rat inner medulla) for two to four days and immunoblotted the samples (Figure 2). After urea incubation, the antibody recognized bovine serum albumin both as a monomer (arrow) as well as in higher molecular weight complexes. In contrast, lanes loaded with samples prepared without urea did not show these bands (Figure 2, left). This result is compatible with the manufacturer's data pointing to specificity of this antibody for carbamylated proteins.

**Figure 3:**
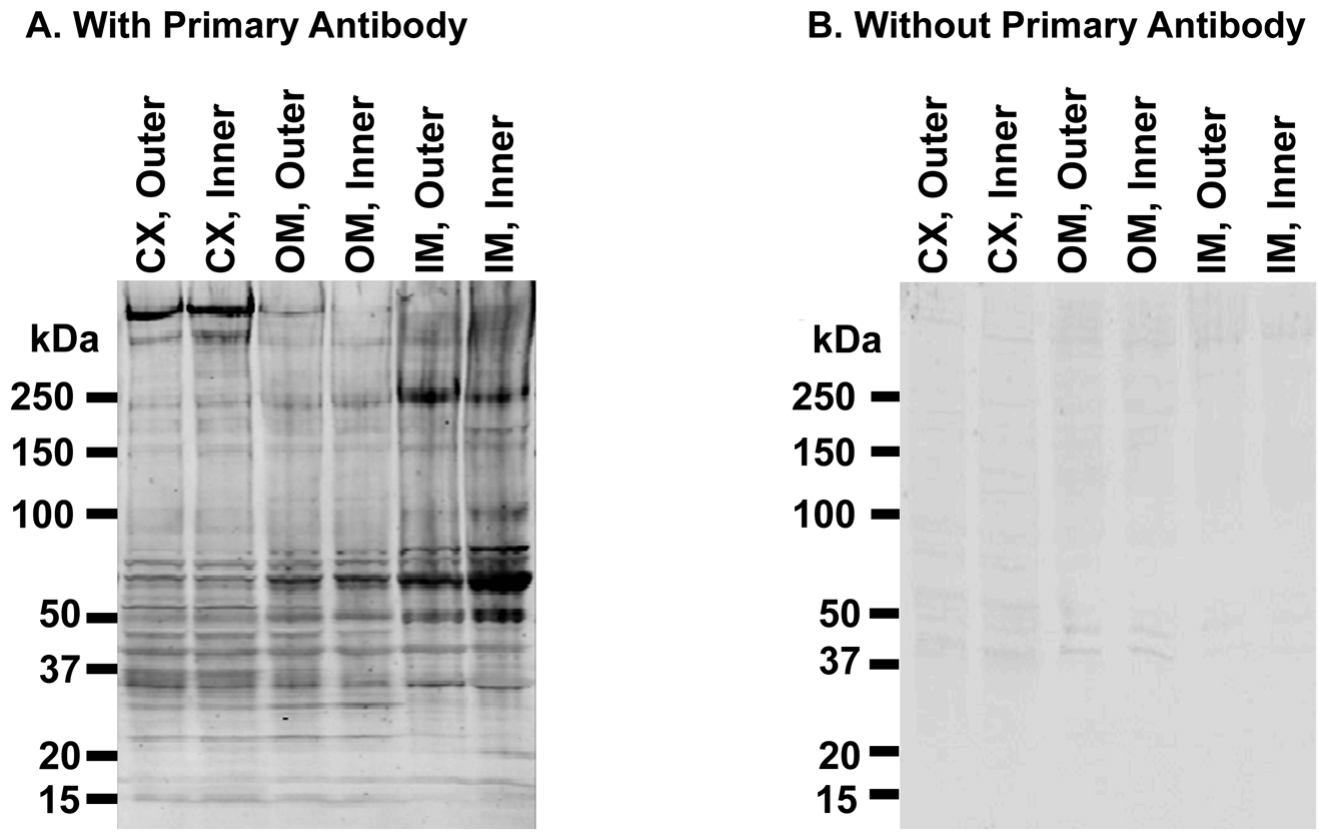
Immunoblots of rat kidney regions with and without the -anti-carbamyl-lysine antibody. A. Immunoblot of rat kidney regions using anti-carbamyl-lysine antibody. B. Control immunoblot of rat kidney regions without primary antibody. Rats were treated with two dDAVP injections (time 0 and 5 hours). 75 ug of protein were loaded onto gels. Abbreviations: CX, cortex; OM, outer medulla; IM, inner medulla. 4–20% polyacrylamide gel.

We used this antibody to probe immunoblots of rat renal cortex, outer medulla, and inner medulla (Figure 3). As hypothesized, many bands were seen in the inner medulla, consistent with the view that multiple carbamylated proteins are present in the inner medulla (Figure 3A). In contrast, no bands were seen in the control immunoblots in which the primary antibody was omitted (Figure 3B). Interestingly, we also observed extensive protein carbamylation in cortex and outer medulla, regions that have much lower physiological levels of urea. This finding is consistent with prior observations that proteins expressed in other organs with low local urea concentrations such as aquaporin-0 [4], αB-crystallin [5], and TIMP-2 [8] can undergo physiological carbamylation in the absence of elevated urea concentrations. Various proteins have also been shown to undergo increases in carbamylation in association with uremic levels of circulating urea [6], [7], [19]–[25].

### Analysis of Protein Carbamylation by Mass Spectrometry

To identify proteins that were endogenously carbamylated in the rat inner medulla, we followed the workflow described in Figure 1. In order to remove urea from the samples prior to trypsinization, we carried out the following procedures: 1) The rats were treated with furosemide 30 minutes prior to sacrifice to reduce the urea content of the inner medulla (Diuretic conditions have been shown to markedly deplete urea from the renal medulla in rats [26]); 2) Medullary samples were dialyzed against a solution that contained no urea but did contain urease to consume urea dialyzed from the tissue; and 3) Samples were separated on SDS-PAGE using urea-free reagents and trypsinization was carried out in the gel pieces cut from these gels.

**Figure 4:**
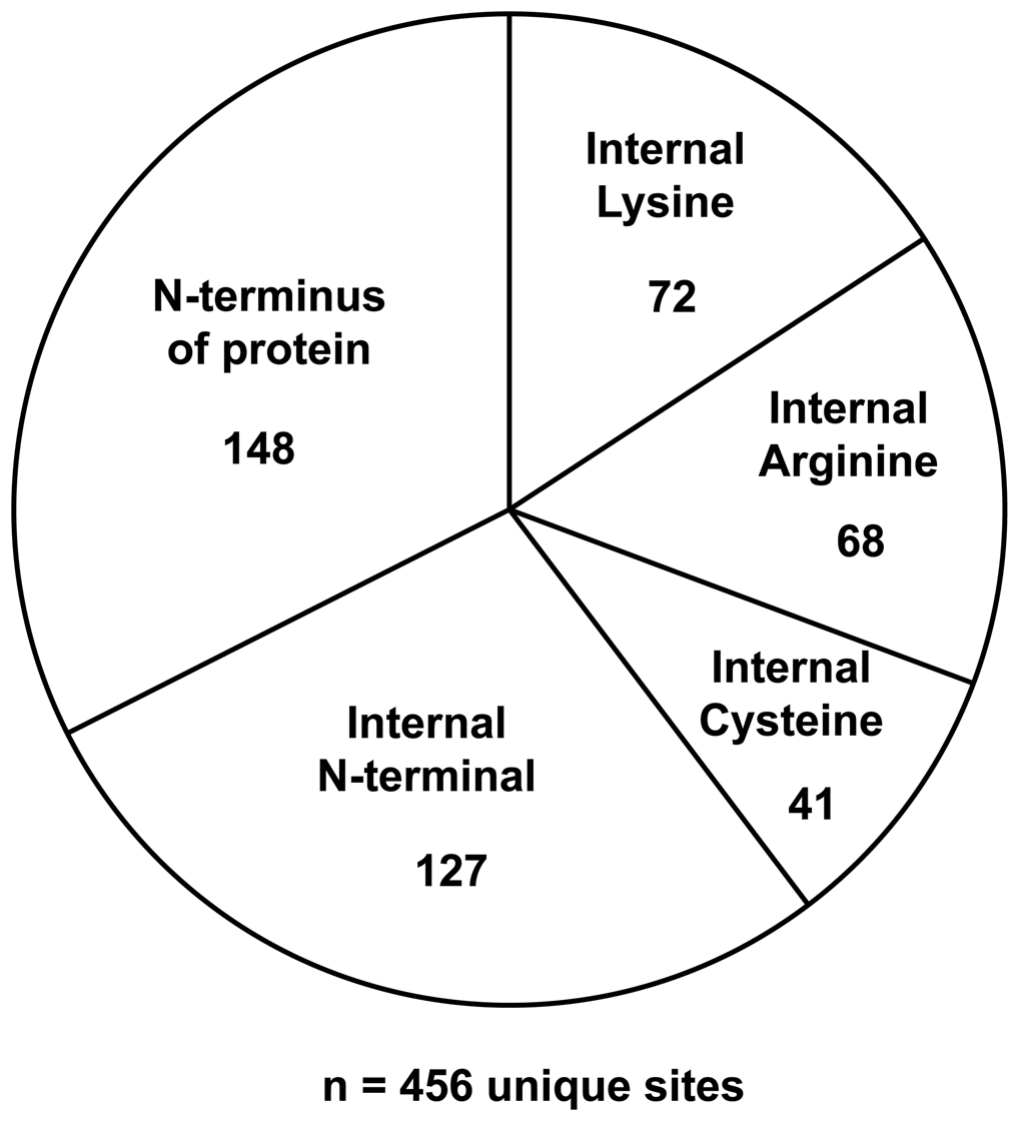
Categories of endogenous carbamylation sites found in inner medullary samples. The carbamylation sites identified are based on pooled mass spectrometry data of carbamyl sites identified in two rats. Rats were treated with furosemide 30

The mass spectrometry analysis of rat inner medulla identified 456 unique carbamylation sites in 403 proteins. Figure 4 shows the distribution of these carbamylation sites by their locations in the proteins. The largest group (approximately one-third of the total) consists of sites that occur at the N-terminal amino acid of the proteins. Smaller fractions include carbamyl-lysines, carbamyl-arginines, and carbamyl-cysteines located in internal portions of the proteins. In addition, a significant number of peptides showed carbamylation of N-termini that are nominally in internal regions of the protein. It is unlikely that these sites are established at the time of trypsinization of the samples because of the exhaustive procedures used to deplete the samples of urea. Indeed, 56 of 127 of these N-terminal sites were carbamylated at nontrypsin sites, i.e., at sites not preceded by lysine or arginine. We propose that these internal sites are present in the intact animal as a result of the action of physiological proteolytic processes, including the protein degradation that occurs as part of normal turnover of proteins in the cell [27]. Note that many proteases in addition to trypsin, e.g. furin, cut after basic amino acids. Therefore, the remaining 71 sites may be targeted by endogenous members of this trypsin-like protease family.

All carbamylated peptides corresponding to Figure 4 are reported at https://helixweb.nih.gov/ESBL/Database/Carbamylation/Carbamylation_peptide_sorted.html. Table 1 shows a subset of the carbamylated proteins chosen based on potential physiological relevance. A wide variety of physiological functions are represented. However, the functional significance of particular carbamylation sites remains unknown.

**Table 1:** Subset of Carbamylated Proteins with Potential Physiological Relevance.

Accession Number	Gene Symbol	Protein Name	Carbamylation Site
NP_036630	Akr1b1	aldose reductase	*A2, C4,*H42, K33, *M13, *S23
NP_077379	Atf4	cyclic AMP-dependent transcription factor ATF-4	*D332, R338
NP_062019	Bsn	bassoon	*L1273
NP_062164	Ca2	carbonic anhydrase 2	*S2
NP_001012054	Calml3	calmodulin-like protein 3	*A2
NP_445809	Ctnnb1	catenin beta-1	*A2
NP_036920	Dlg1	disks large homolog 1	*E561
NP_001013122	Eef1d	elongation factor 1-delta	*A371, *E619
NP_001099307	Eif1	eukaryotic translation initiation factor 1	*S2
NP_001094012	Eif2s3x	eukaryotic translation initiation factor 2 subunit 3	*A2
NP_036686	Eno1	alpha-enolase isoform 1	*L163
NP_068630	Gnb3	guanine nucleotide-binding protein (G protein), beta-3	C317
NP_001013932	Gnb4	guanine nucleotide-binding protein (G protein), beta-4	C294
NP_113958	Gnb5	guanine nucleotide-binding protein (G protein), beta-5	*A2
NP_001244278	Gng2	guanine nucleotide-binding protein (G protein), gamma 2	*A2
NP_036866	Hk1	hexokinase-1	M1, *I163
NP_001004082	Hsp90ab1	heat shock protein HSP 90-beta	*E492
NP_058721	Ldha	L-lactate dehydrogenase A chain	*A2, *A3
NP_063969	Lgals1	galectin-1	*A2
NP_037326	Myh9	myosin-9	*A2, *M941, *D1705
NP_598267	Ndrg2	NDRG2	*A2
NP_001030103	Pkhd1l1	fibrocystin-L precursor	*V428
NP_001128261	Polr2h	polymerase (RNA) II (DNA directed) polypeptide H	*A2
NP_001178807	Polr3b	DNA-directed RNA polymerase III subunit RPC2	C769
NP_071943	Ppp1cc	serine/threonine-protein phosphatase PP1-gamma catalytic subunit	*A2
NP_001178875	Ppp1r12c	protein phosphatase 1 regulatory subunit 12C	*S2
NP_062137	Prkar2a	cAMP-dependent protein kinase type II-alpha regulatory subunit	*S2
NP_058980	Psmb2	proteasome subunit beta type-2	M1
NP_112411	Psmc5	26S protease regulatory subunit 8	*A2
NP_599193	Rac1	ras-related C3 botulinum toxin substrate 1 precursor	*P50
NP_001157736	Rnf187	E3 ubiquitin-protein ligase RNF187	K170
NP_114467	Ryr2	ryanodine receptor 2 isoform 1	R1024
NP_445937	S100a6	S100-A6	*A2, C3
NP_114025	Sept9	septin-9 isoform 1	*S2
NP_001012103	Trim32	E3 ubiquitin-protein ligase TRIM32	*A2
NP_001100525	Wnt9b	Wnt-9b precursor	C57, K61

N-terminus of peptide.

**Figure 5:**
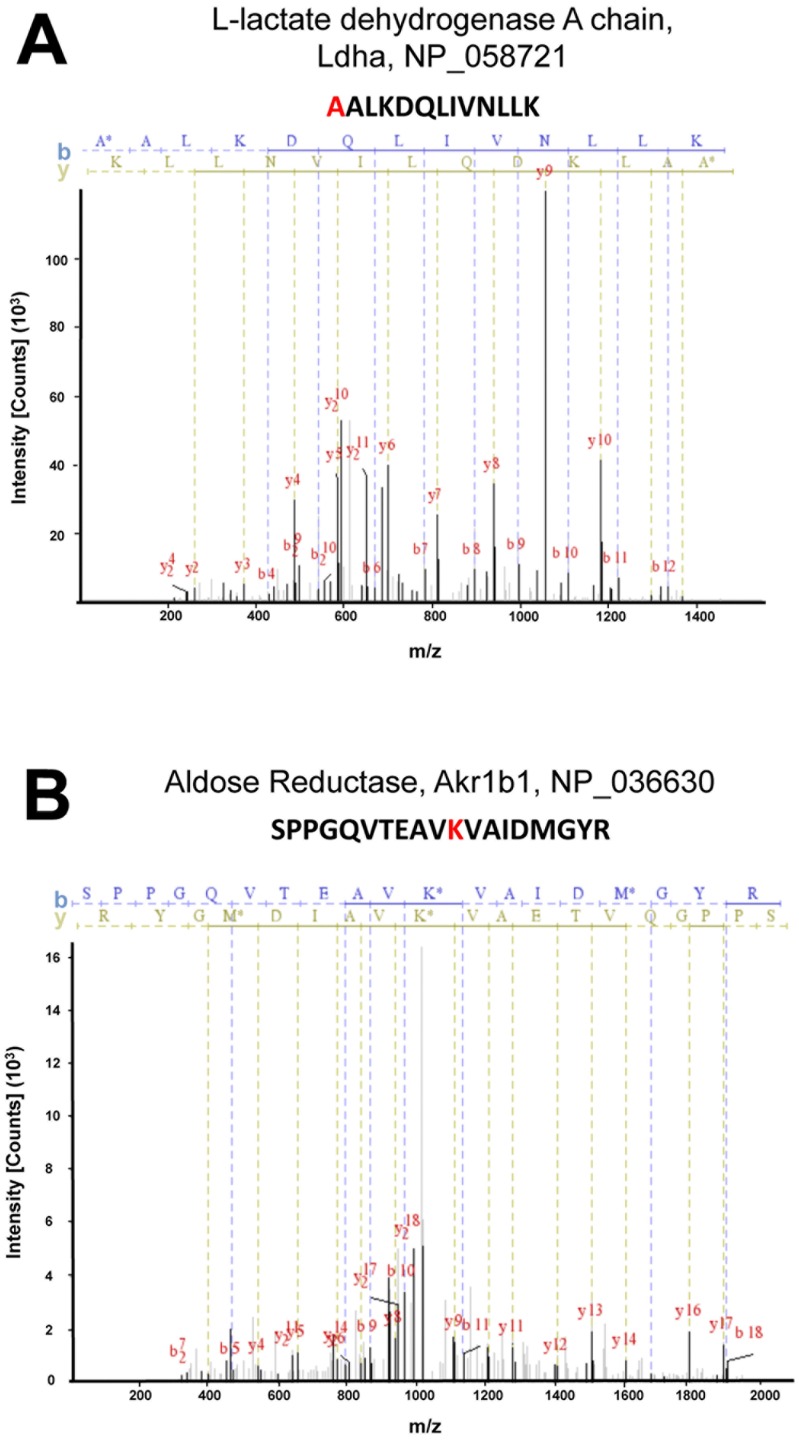
Example spectra of carbamylated peptides. A. Example of N-terminal carbamylation: L-lactate dehydrogenase A chain (Gene Symbol: Ldha; Accession number: NP_445937). Matched ions are denoted by a semi-continuous vertical line (blue for b-ions and brown/green for y-ions). All y-ions show the additional 43.005814 Da mass indicative of a carbamyl group. B. Example of carbamyl-lysine spectrum: Aldose Reductase (Gene Symbol: Akr1b1; Accession number: NP_036630)). All y-ions including and above y^9^ show the additional 43.005814 Da mass indicative of a carbamyl group. Images are from InsPecT (See *Methods*).

Figure 5 shows two examples of collision-induced dissociation fragmentation spectra of carbamylated peptides found in this study. Figure 5A shows an N-terminal carbamylation site in L-lactate dehydrogenase A chain (*Ldha*). In the spectrum, carbamylation is inferred by the presence of the added mass of a carbamyl group (minus -H) in all fragments that contain the original N-terminus. Figure 5B shows carbamylation of an internal lysine present in aldose reductase (*Akr1b1*), a protein involved in osmotic regulation in the renal inner medulla [28]. In the spectrum, carbamylation is inferred by the presence of the added mass of a carbamyl group (minus -H) in all detectable peptide fragments that contain K33, the carbamylated lysine. Figure S1 displays six additional example spectra showing carbamylation of physiologically important proteins. In addition, the reader can view the spectra of all identified carbamylation sites online at https://helixweb.nih.gov/ESBL/Database/Carbamylation/Carbamylation_peptide_sorted.html.

**Figure 6:**
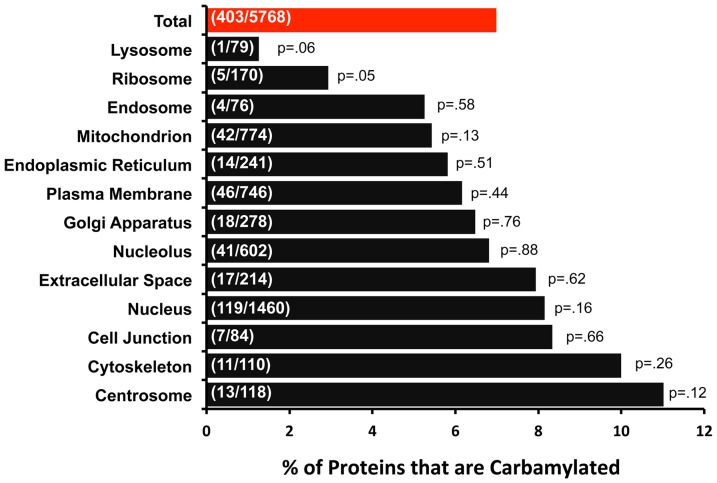
Gene Ontology Cellular Component analysis reporting percent of proteins identified in various cellular structures that were found to be carbamylated. The overall total is shown as a red bar. Fraction in bars show number of carbamylated/(carbamylated+non-carbamylated) proteins. A total of 5768 proteins were detected in all samples counting proteins with both carbamylated and non-carbamylated peptides. The *P* values were obtained using a chi-squared test versus “Total”.

Next, we asked whether carbamylation is characteristic of any particular subcellular compartment. To address this, we assigned *Gene Ontology Cellular Component* terms to all carbamylated and noncarbamylated proteins detected in the study. The results are summarized in Figure 6. Overall, slightly more than seven percent of identified proteins were found to be carbamylated (red bar) and all compartments are represented. We speculate that the lower fraction of lysosomal proteins that are carbamylated may be because of the low pH inside of lysosomes, which would titrate the cyanate ion (the reactive form) to cyanic acid.

What could be the physiological role of carbamylation, if any? First, like acetylation, carbamylation may alter the function of a given protein by modifying the amino acid charge, causing positively charged moieties to become neutral [29]. This could alter the protein folding or its ability to interact with other macromolecules. Another possibility is that carbamylation of lysines, arginines, cysteines, and protein N-termini may prevent other post-translational modifications. For example, lysines undergo multiple post-translational modifications including sumoylation, ubiquitylation, and acetylation. These can have important consequences with regard to the function and stability of the target protein. For example, ubiquitylation is known to mark proteins for degradation either by the proteasome pathway or via lysosomes. Table 2 shows a number of carbamylation sites found in this study that have been previously identified as ubiquitylation sites (PhosphoSite Plus ubiquitylation database, http://www.phosphosite.org/homeAction.do).

**Table 2:** Identified carbamylation sites that have been previously shown to be ubiquitylated according to *PhosphoSite Plus* database.

Accession Number	Gene Symbol	Protein Name	Carbamylation Site
NP_112266	Acta2	actin, aortic smooth muscle	K293
NP_062056	Actc1	actin, alpha cardiac muscle 1	K293
NP_037025	Actg2	actin, gamma-enteric smooth muscle	K292
NP_599153	Alb	serum albumin precursor	K257
NP_037289	Ass1	argininosuccinate synthase	K165
NP_001020866	Cops5	COP9 signalosome complex subunit 5	K54
NP_001102870	Hist1h2bc	H2bm, histone H2B	K58
NP_001101884	Hist1h2bm	H2ba, histone cluster 1	K58
NP_001104597	Hist3h2ba	histone cluster 3	K58
NP_001103111	Hist3h2bb	histone cluster 3, H2bb	K58
NP_077327	Hspa8	heat shock cognate 71 kDa protein	K561
NP_569105	Lcp2	lymphocyte cytosolic protein 2	K253
NP_001157736	Rnf187	E3 ubiquitin-protein ligase RNF187	K170
NP_741984	Sptan1	spectrin alpha chain, brain	K1519

### Effects of Water Balance Changes

**Figure 7:**
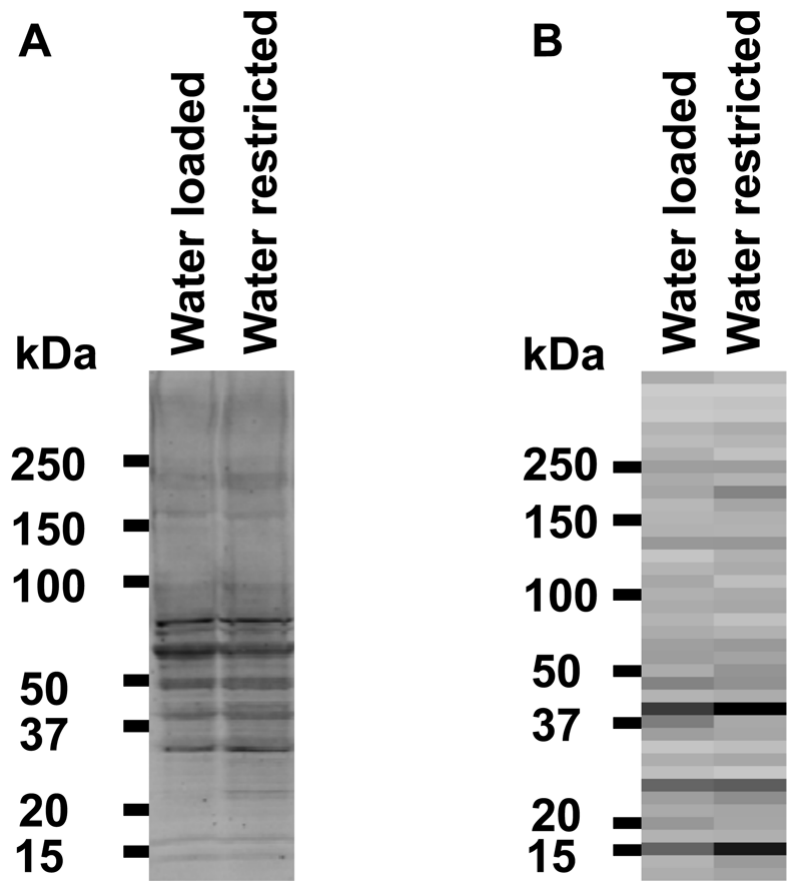
Effects of water balance changes on carbamylation. A. Immunoblot of Water Loaded rat vs. Water Restricted rat using anti-carbamyl-lysine antibody. Animals were water restricted (17 ml of water/day) or water loaded (40 ml of water/day) for five days. B. Heat map representation of mass spectrometry data provides a pseudo immunoblot of total carbamylated proteins. Animal treatments were identical to those used in A.

**Figure 8:**
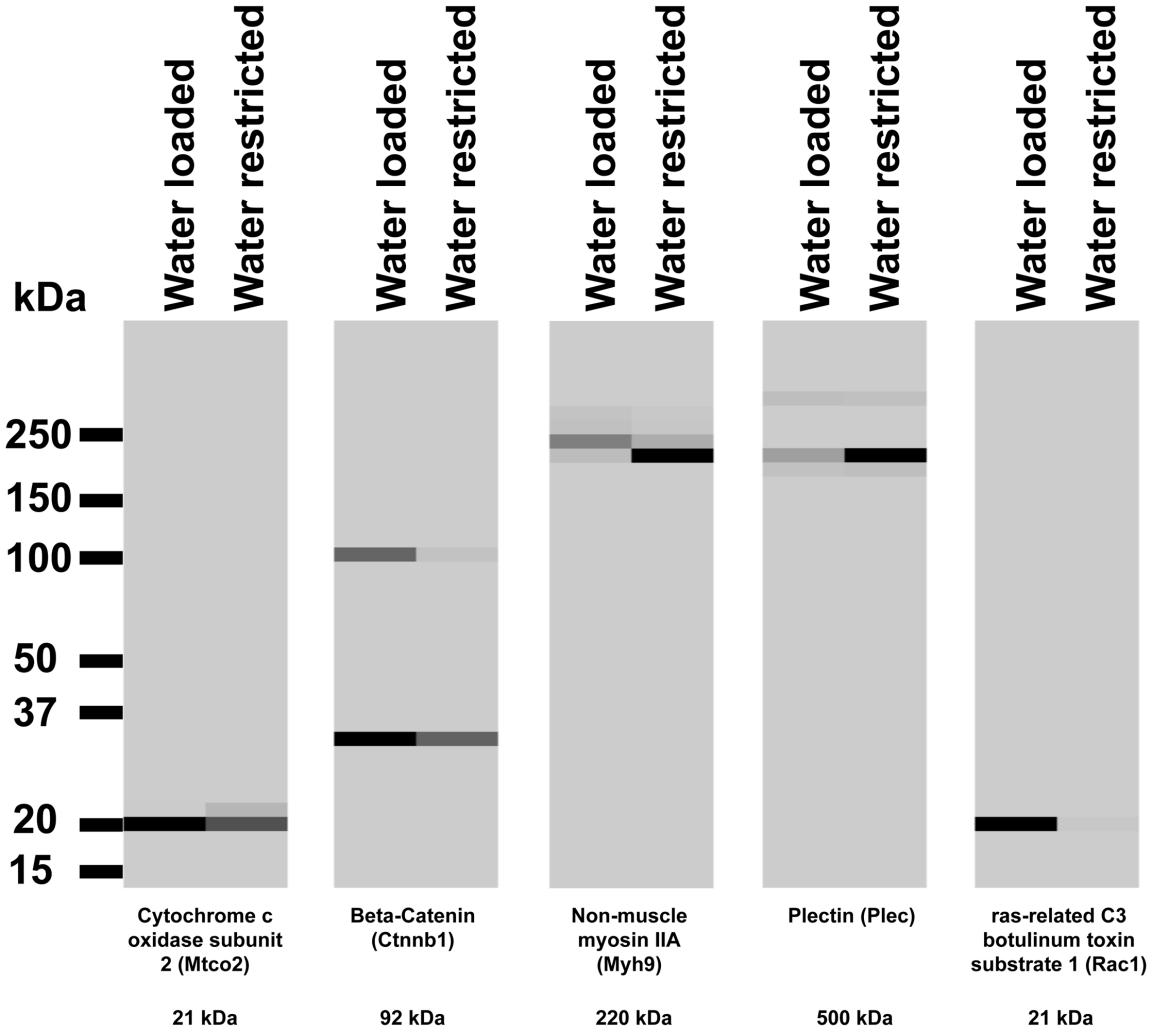
Heat map representation of mass spectrometry data provides a pseudo immunoblot of five carbamylated proteins. For a given protein, the pseudo immunoblot shows carbamylated peptides found in each treatment group (Water Loaded vs Water Restricted) according to molecular weight and relative abundance. Molecular weights given are theoretical, based on known amino acid sequence.

Figure 7 shows experiments comparing the effects of water loading for 72 hours with water-restriction for 72 hours (n = 3 rats for both). Water-restriction does not significantly increase the amount of total protein carbamylated when compared to water loading. Figure 7B shows a heat map representation of mass spectrometry data in the form of a pseudo western blot. This figure shows the distribution of all carbamylated peptides with regard to molecular weight. Again, there was no clear effect of water balance changes on carbamylation of rat inner medulla proteins. Figure 8 shows the distribution of carbamylated peptides as a function of molecular weight of several individual proteins. In general the molecular weight inferred from the mass spectrometry studies agrees with the nominal molecular weight of the individual proteins. In addition, lower molecular weight forms were identified for plectin and β-catenin. The identified 240 kDa fragment of plectin corresponds to a known physiological cleavage product [30]. β-catenin is known to undergo physiological cleavage as part of the Wnt signaling pathway [31].

### Conclusions

We conclude from these observations that protein carbamylation occurs endogenously in the kidney and is present in multiple proteins that play important physiological roles. However, contrary to the hypothesis expressed in the Introduction, protein carbamylation does not appear to be restricted to the inner medulla where urea is highest in the kidney (Fig. 3). Nor is there a striking difference in carbamylation in the inner medulla as a result of variation in water intake (Fig. 7), which is associated with striking changes in levels of tissue urea in the renal inner medulla [10]. It seems likely that circulating levels of urea, typically 4 mM in nondiuretic rats [32], is sufficient to result in carbamylation, providing an explanation for previous findings that several non-renal proteins can be carbamylated (see Introduction).

## Supporting Information

Figure S1 **Additional example spectra of carbamylated peptides.** Six example spectra showing carbamylation of physiologically important proteins.
(TIF)
