# Supplementary figures and images for: Endogenous Carbamylation of Renal Medullary Proteins
Source: PLoS One. 2013 Dec 26;8(12):e82655. doi: 10.1371/journal.pone.0082655 (PMC3873256; doi:10.1371/journal.pone.0082655)

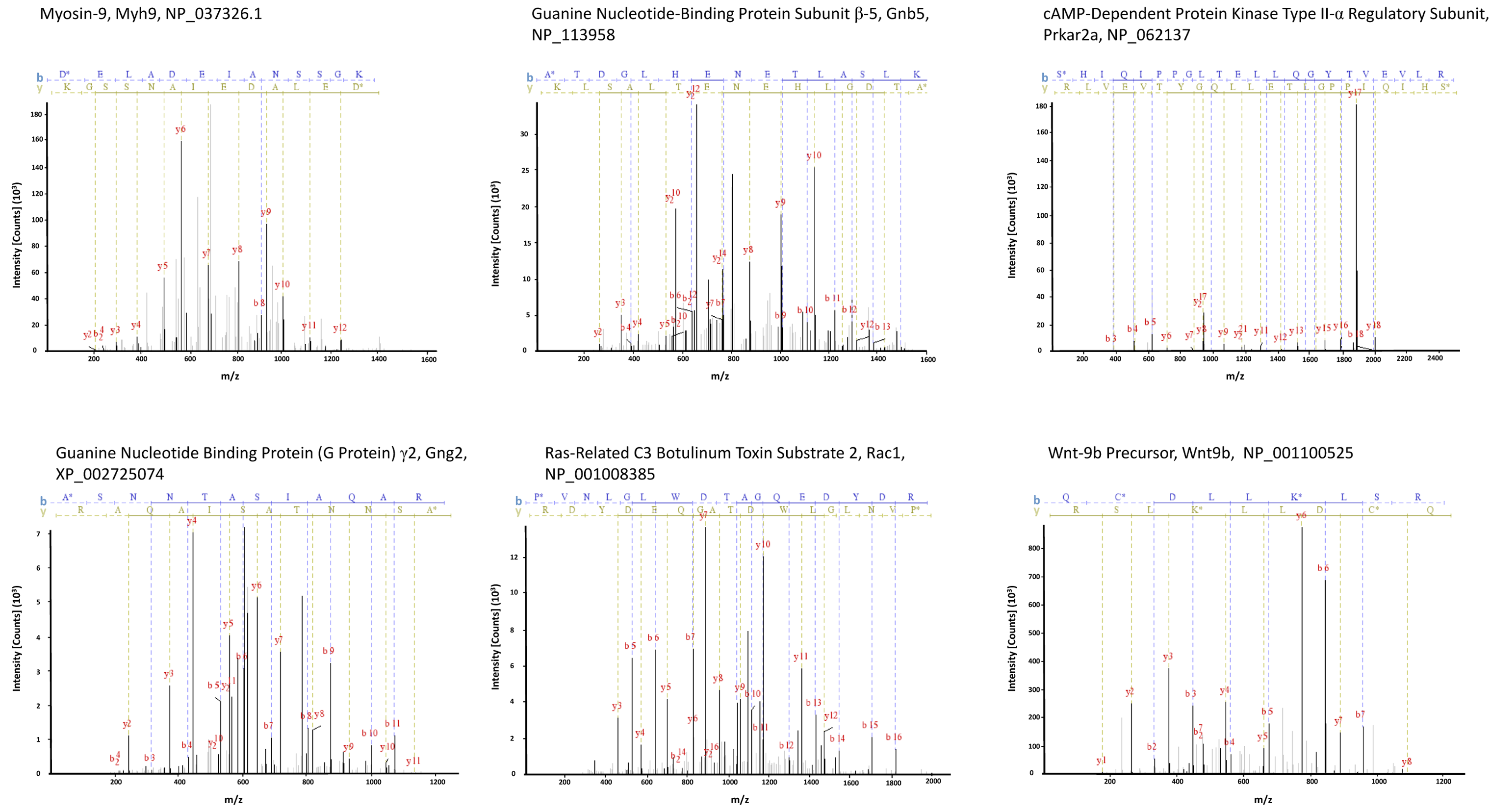

Supplement: Figure S1 — Additional example spectra of carbamylated peptides. Six example spectra showing carbamylation of physiologically important proteins. (TIF) [file pone.0082655.s001.tif]
